# Glycyrrhizic Acid Prevents Diabetic Nephropathy by Activating AMPK/SIRT1/PGC-1*α* Signaling in db/db Mice

**DOI:** 10.1155/2017/2865912

**Published:** 2017-11-07

**Authors:** Shaozhang Hou, Ting Zhang, Yuan Li, Fengying Guo, Xiu Jin

**Affiliations:** ^1^Department of Pathology, School of Basic Medical Sciences, Ningxia Medical University, Yinchuan 750004, China; ^2^Institute of Basic Medical Sciences, Ningxia Medical University, Yinchuan 750004, China; ^3^Department of Nursing, Ningxia Medical University, Yinchuan 750004, China; ^4^Affiliated Hospital of Jining Medical College, Jining 272000, China

## Abstract

Diabetic nephropathy (DN) is a major cause of end-stage renal disease (ESRD). Glycyrrhizic acid (GA) is an effective inhibitor of reactive oxygen species (ROS) production. We investigated the role of GA in the progression of renal injury in DN. Albumin (Alb)/creatinine (crea) levels were significantly lower, and renal histopathology was attenuated in the diabetic db/db mice that were treated with GA (15 mg/kg via intraperitoneal injection) once per day for eight weeks. These changes were associated with significantly lower levels of *α*-smooth muscle actin (*α*-SMA) and transforming growth factor *β*1 (TGF-*β*1) expression. Additionally, diabetic db/db mice displayed more terminal deoxynucleotidyl transferase-mediated nick-end labeling- (TUNEL-) positive nuclei and diabetes-induced ROS production in the kidneys, and these effects were attenuated by the treatment with GA, which activated adenosine monophosphate-activated protein kinase (AMPK)/silent information regulator 1 (SIRT1)/peroxisome proliferator-activated receptor-*γ* coactivator-1*α* (PGC-1*α*) signaling in the kidneys. In summary, in diabetic db/db mice, the effect of GA on DN involved, in part, the inhibition of ROS and the activation of AMPK/SIRT1/PGC-1*α* signaling in the kidneys. GA, therefore, shows therapeutic potential for preventing and treating DN.

## 1. Introduction

Diabetic nephropathy (DN) is a major cause of end-stage renal disease (ESRD) and one of the most severe microvascular complications of both type 1 and type 2 diabetes [1–4]. In diabetic patients, the pathogenesis and progression of chronic kidney disease (CKD) involve numerous factors, such as hyperglycemia-induced oxidative stress and inflammation. Hemodynamic changes and disorders of glucose metabolism can be caused by genetic factors, hyperglycemia, or changes in cytokine levels and can result in the development of DN [2, 5, 6]. Although chronic exposure to elevated glucose levels is a major cause of kidney disease, the mechanisms that lead to the development and progression of kidney injury in these patients remain unclear [7, 8]. It is therefore important to explore new approaches aimed at preventing and treating DN.

Glycyrrhizic acid (GA) is a triterpenoid saponin glycoside that is known as the most efficacious component of the licorice plant [9, 10]. Previous studies have shown that GA exhibits antiulcerative, expectorant, antiviral, anti-inflammatory, antidiabetic, anticancer, neuroprotective, and immune-enhancing properties [11, 12]. Additionally, GA has been shown to alleviate benzopyrene-induced lung injury [13], to exert a therapeutic effect on asthma by regulating immune functions [14], and to prevent renal ischemia-reperfusion injury in rabbits [15]. Furthermore, it is widely accepted that the effects of GA are mediated by both SIRT1 and AMPK [16]. However, the role that GA plays in the kidneys of type 2 diabetic animals has not been explored.

In this study, we investigated the protective effect exerted by GA against oxidative stress and found that GA activates AMPK/SIRT1/PGC-1*α* signaling in diabetic db/db mice. These results increase our understanding of the mechanisms underlying DN. Furthermore, these data suggest targets for the search for a potential therapy for DN.

## 2. Materials and Methods

### 2.1. Materials

GA was obtained from Tokyo Chemical Industry Co. (TCI, Tokyo, Japan). A reactive oxygen species assay kit was purchased from Beyotime (Jiangsu, China). AMPK*α*, p-AMPK*α*, SIRT1, PGC-1*α*, TGF-*β*1, and *α*-SMA antibodies were purchased from Abcam. *β*-actin was purchased from Bios (Beijing, China).

### 2.2. Animals and Grouping

Male db/db (BKS.Cg-m+ *Lepr*^db^/+ Lepr^*db*^/Jcl) and db/m (BKS.Cg-m +/+Lepr db/Jcl) mice were purchased from Mode Animal Research Center of Nanjing University. The mice were maintained in a specific pathogen-free colony in the Laboratory Animal Center of Ningxia Medical University (Yinchuan, China) in facilities with controlled temperature and humidity, and 12 : 12 hour light and dark cycle. The body weight and fasting blood glucose level were monitored weekly. The study was conducted in accordance with the “Guide for the Care and Use of Laboratory Animals” and approved by the Institutional Animal Care and Use Committee of Ningxia Medical University.

Eight-week-old male mice were divided into the following five different groups, which received either 0.9% sodium chloride (NaCl) or GA: db (db/db mice treated with NaCl, *n* = 10), db + g (db/db mice treated with GA, *n* = 10), m (db/m mice treated with NaCl, *n* = 10), m + g (db/m mice treated with GA, *n* = 10), and n (normal group, C57BL/6 mice treated with NaCl, *n* = 10. C57BL/6 mice is a common inbred strain of laboratory mouse). The groups administered with GA received a dose of 15 mg/kg via an intraperitoneal (i.p.) injection once per day for eight weeks starting when the mice were eight weeks old. The db, m, and n group mice were administered (i.p.) with 0.9% sodium chloride at the same time points. The mice were euthanized to remove the kidneys. The kidneys were rapidly fixed in normal-buffered 4% paraformaldehyde for immunohistochemical analyses, and all experiments were performed using renal cortex samples. Blood was collected from the left ventricle, and plasma was stored at −80°C.

### 2.3. Histological Analysis

Paraformaldehyde-fixed and paraffin-embedded renal tissues were sectioned (4 *μ*m thickness) and stained with hematoxylin and eosin (HE) and periodic acid-Schiff (PAS). For the electron microscopic studies, tissue sections were dehydrated through an ascending series of ethanol (to 100%) and then washed in dry acetone and embedded in epoxy resin. After the sections were fixed, dehydrated, embedded, and sliced, they were stained with uranyl acetate. Ultrathin sections were counterstained with lead citrate and then examined using transmission electron microscopy (H7650).

### 2.4. Immunohistochemistry Assay

The protein levels of AMPK*α*, p-AMPK*α*, SIRT1, PGC-1*α*, TGF-*β*1, and *α*-SMA (1 : 200, Abcam, USA) were determined in each group by incubating the samples in primary antibodies overnight followed by incubation with secondary antibodies and IHC detection reagent (ZSGB-BIO, Beijing, China) at 37°C for 45 min. The results were detected using DAB (ZSGB-BIO, Beijing, China), and hematoxylin staining was used to label the nuclei. The cells were observed and photographed using an optical microscope (BX61). The number of positively stained cells was counted in five microscopic fields at 400x.

### 2.5. Western Blot Analysis

Protein was isolated from tissues, and the concentration of proteins in each sample was determined to ensure that the sample volumes were consistent. Equal amounts (50 *μ*g) of protein extracts were subjected to 10%–15% sodium dodecyl sulfate-polyacrylamide gel electrophoresis (SDS-PAGE) and then transferred to polyvinylidene fluoride membranes (Millipore). The membranes were incubated overnight at 4°C with the following primary antibodies: anti-AMPK*α* (1 : 1000), anti-p-AMPK*α* (1 : 1000), anti-SIRT1 (1 : 1000), anti-PGC-1*α* (1 : 1000), anti-TGF-*β*1 (1 : 2000), anti-*α*-SMA (1 : 2000), and anti-*β*-actin (1 : 3000). The membranes were then incubated with horseradish peroxidase-conjugated secondary antibodies for 60 min at room temperature. Imaging was performed using a Bio-Rad imaging system with chemiluminescence detection reagents. The densitometry of the bands was assessed using Image-Pro Plus 6.0 software.

### 2.6. ROS Detection

Frozen renal cortex sections were cultured and incubated in the dark with 10 *μ*m/L of dihydroethidium (DHE) for 30 min at 37°C. Intracellular ROS production was assessed using an Olympus FluoView1000 confocal laser scanning microscope (using Ex/Em *λ* = 480 nm/535 nm for DHE).

### 2.7. TUNEL Staining

Terminal deoxynucleotidyl transferase-mediated dUTP nick-end labeling (TUNEL) staining was used to detect apoptosis. Apoptotic cells were examined using a TdT DNA fragmentation detection kit (Roche, Mannheim, Germany) according to the manufacturer's protocol. The percentage of apoptotic cells was calculated as the ratio of the number of TUNEL-positive cells to the total number of cells.

### 2.8. Statistical Analysis

All quantitative data are expressed as mean ± SD. Differences between the two experimental conditions were assessed using Tukey's test. The statistical significance of these differences was analyzed using one-way ANOVA, and a value of *p* < 0.05 was considered statistically significant.

## 3. Results

### 3.1. Effect of GA on the Onset of DN in db/db Mice

**Figure 1:**
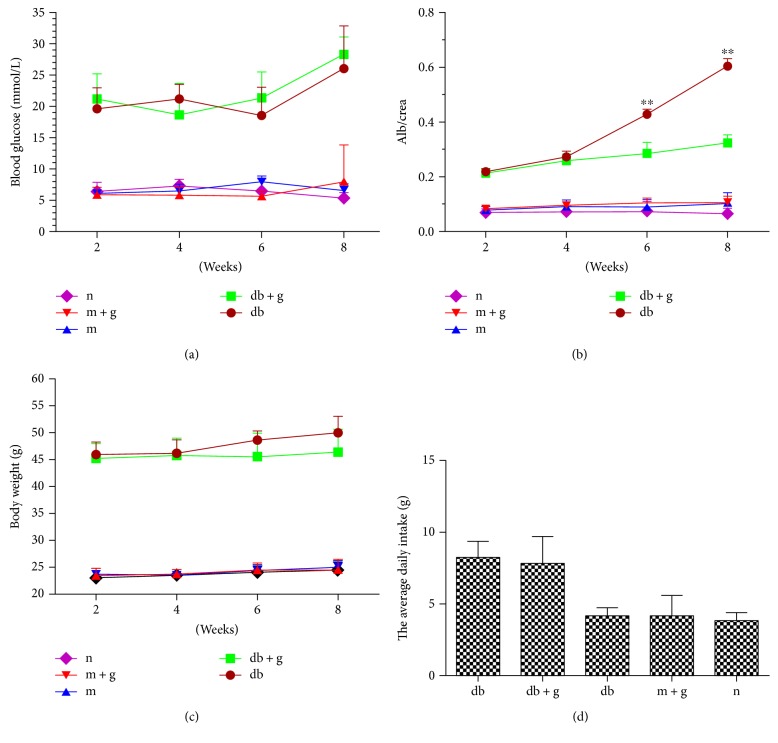
Effect of GA on the onset of DN in db/db mice. Treatment with GA did not significantly alter blood glucose levels (a), body weight (c), or food intake (d) in db/db mice. Alb/crea levels were significantly lower in the db + g group than in the db group (^∗∗^*p* < 0.05 versus db + g).

Blood glucose levels, body weight, and food intake were significantly higher in db/db mice than in the other groups. Treatment with GA did not significantly alter blood glucose levels, body weight, or food intake in db/db mice. Alb/crea levels were significantly higher in the db group than in the other groups, and treatment with GA for 8 weeks significantly decreased Alb/crea levels in the db + g group but not in the db group (Figure 1).

### 3.2. Histopathological Findings in db/db Mice Treated with GA

**Figure 2:**
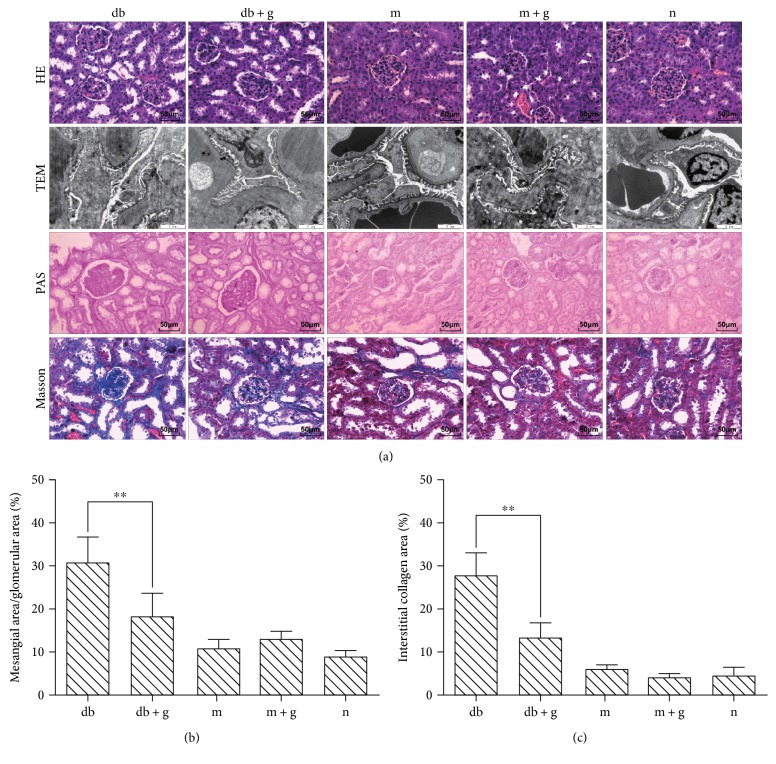
Effect of GA on kidney pathology in db/db mice. Representative sections obtained from the kidneys of mice (8 weeks old) in each group were stained with (a) HE ×400, electron microscopy (TEM) ×2000, and PAS ×400, Masson staining (blue) ×400. (b) Quantitative estimates of the mesangial fractional area (%). (c) Quantitative assessment of the interstitial collagen area (%). Values are shown as means ± SD. ^∗∗^*p* < 0.01.

Mesangial expansion is a characteristic finding in diabetic glomerulopathy (Figure 2). HE staining revealed that the glomerular structure was normal, and there was no clear difference in proliferation in glomerular mesangial cells between the db and normal control group mice. Mesangial expansion, glomerular hypertrophy, and mesangial matrix gathering were clearly observed in the db/db mice, and these symptoms were substantially ameliorated by treatment with GA. The mesangial area was determined using PAS staining and found to be higher in the db/db mice than in the db/m and wild-type mice. Electron microscopy revealed the capillary ultrastructure of the glomerulus. Electron micrographs confirmed that in the db/db mice, mesangial volume was higher, glomeruli were irregularly thickened, and the basement membranes exhibited splitting. Treatment with GA produced clear changes. For example, basement membrane thickness was significantly decreased. Masson staining was used to dye collagen fibers blue, and the db/db group mice exhibited heavier renal fibrosis than that observed in the db + g group mice. We found that collagen fibers had significantly accumulated in the db/db group. Consistent with the changes observed in the glomerulus, treatment with GA markedly reduced the increase in collagen deposition that was observed in the renal interstitium in the db/db mice (assayed using Masson staining as shown in Figures 2(b) and 2(c)). These results indicate that treatment with GA may attenuate diabetic renal interstitial fibrosis *in vivo.*

### 3.3. GA Reduces Apoptosis in Renal Cells in db/db Mice

**Figure 3:**
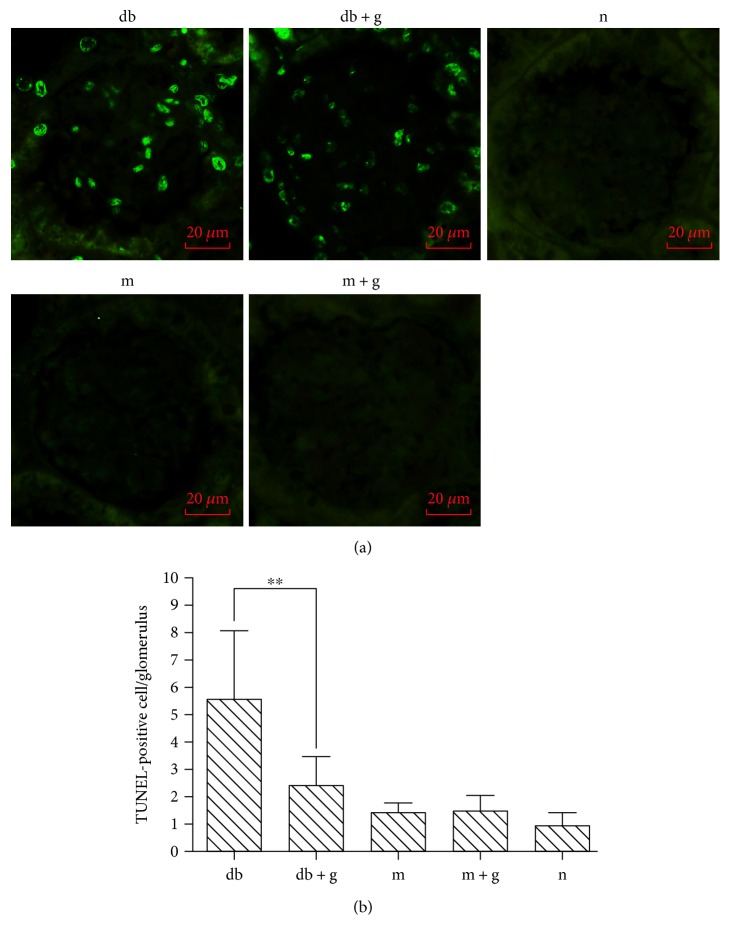
Effect of GA on apoptosis of cells in the renal cortex of db/db mice. Representative micrographs of TUNEL staining in the renal cortex (a). The same data are shown as a graph (b). The graph shows the number of TUNEL-positive cells that were detected in the glomerulus. Original magnification, ×400. ^∗∗^*p* < 0.001 compared with the control group.

To assess cellular apoptosis in diabetic kidneys, renal tissue sections were submitted to a TUNEL assay. In 8-week-old mice, there were more apoptotic cells in the diabetic db/db mice than in the db/m mice. There were fewer apoptotic cells in the db/db mice treated with GA than in the corresponding db group (Figure 3). These data demonstrate that treatment with GA attenuates diabetes-induced apoptosis in type 2 diabetic kidneys.

### 3.4. Effect of GA on the Expression of *α*-SMA and TGF-*β*1 in db/db Mice

**Figure 4:**
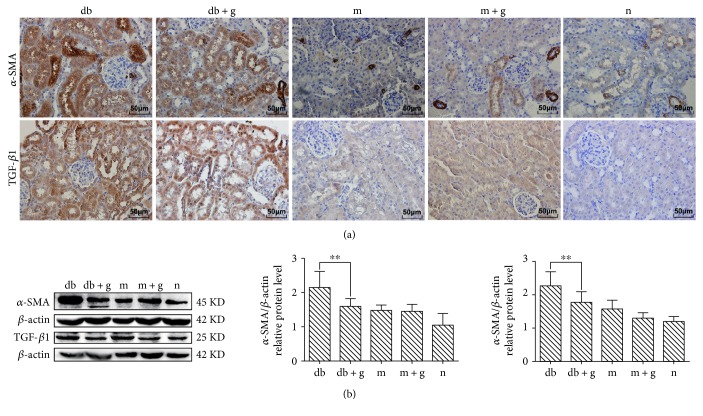
Effect of GA on the expression of *α*-SMA and TGF-*β*1 in the renal cortex of db/db mice. (a) Immunohistochemistry was used to detect the expression of *α*-SMA and TGF-*β*1. Original magnification: ×400. (b) Western blot assays were used to detect *α*-SMA and TGF-*β*1 in mice treated with GA. The proportion of cells with positive protein expression is shown. Histograms indicate the mean ± SD (^∗∗^*p* < 0.05). *β*-actin was detected to confirm equal loading. The gray graph indicates the relative level of protein expression in each group.

*α*-SMA and TGF-*β*1 have been shown to stimulate proliferation in mesangial cells and increase the synthesis of extracellular matrix (ECM), and both have been identified as key mediators of glomerular and tubulointerstitial pathologies in chronic renal diseases. Immunohistochemical and western blot analyses showed that DN increased *α*-SMA and TGF-*β*1 expression (Figure 4), and both were expressed at significantly lower levels in the db + g and db control groups (*p* < 0.05).

### 3.5. Effects of GA on Diabetes-Induced ROS Production in the Kidneys of db/db Mice

**Figure 5:**
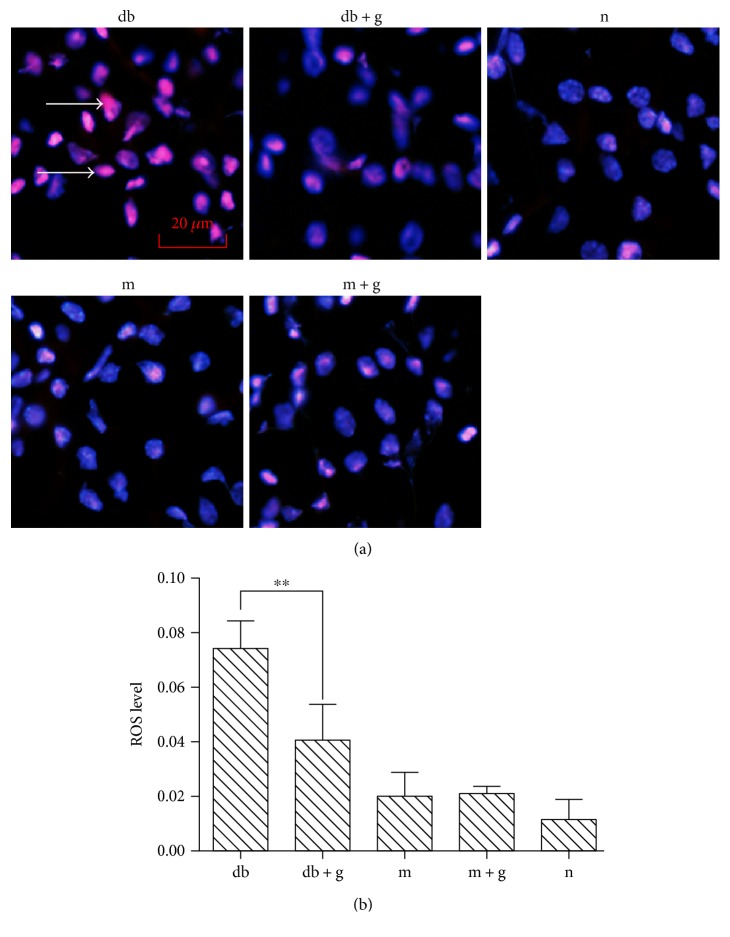
Effect of GA on ROS levels in the kidneys of db/db mice. The bar graph demonstrates the increases in ROS production observed in the renal cortex of db/db mice and the antioxidant effect of GA. The lower panels are representatives of the original recorded data. The data were collected from 3 independent experiments and are presented as means ± SD. ^∗∗^*p* < 0.05 compared with the control. One-way ANOVA followed by Tukey's test.

ROS production was measured using a DHE probe that detects superoxide. As shown in Figure 5(a), DN significantly enhanced the production of ROS in db/db mice, and pretreatment with GA prevented this DN-induced increase in ROS (*p* < 0.05). Thus, the levels of ROS were the same in the normal group, the m group, and the m + g group (*p* > 0.05). In this study, the DN-induced production of ROS was augmented in the db group but decreased in the db + g group. These data indicate that the administration of GA either impaired the generation of ROS or increased the capacity of the cell to perform endogenous ROS scavenging/antioxidant activities.

### 3.6. Effect of GA on p-AMPK*α*, SIRT1, and PGC-1*α* Expression in db/db Mice

**Figure 6:**
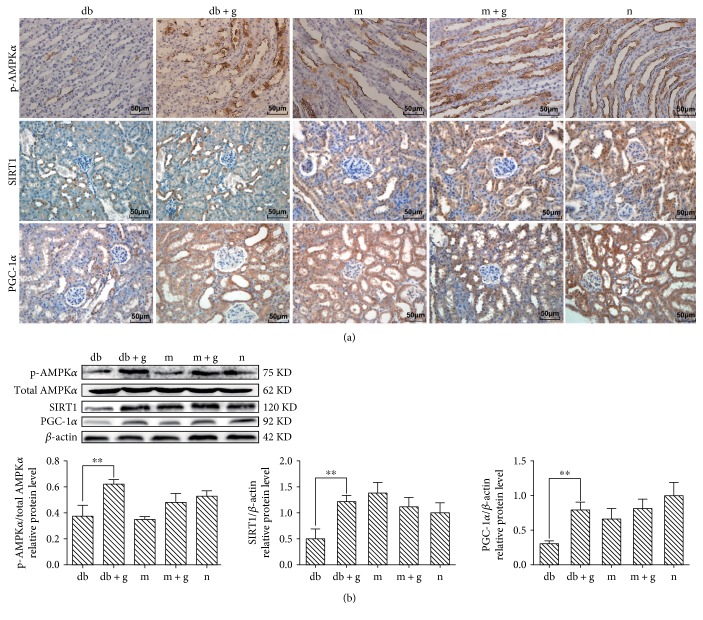
Effect of GA on p-AMPK*α*, SIRT1, and PGC-1*α* expression in the kidneys of db/db mice. (a) Immunohistochemistry was used to detect p-AMPK*α*, SIRT1, and PGC-1*α* in mice treated with GA. Original magnification: ×400. (b) Western blot analysis was used to detect specific proteins, and the proportion of cells with positive protein expression was determined. Histogram values represent the mean ± SD (^∗∗^*p* < 0.05). *β*-actin was detected to confirm equal loading. The gray graphs show the relative protein levels in each group.

In this study, we used immunohistochemistry and western blot analyses to assess the effect of GA on p-AMPK*α*, SIRT1, and PGC-1*α* protein expression levels (Figure 6). GA administration caused the expression of p-AMPK*α*, SIRT1, and PGC-1*α* to be higher in the db + g group than in the db group (*p* < 0.05). These results indicate that AMPK/SIRT1/PGC-1*α* signaling is induced in the kidneys of db/db mice and that GA effectively prevents DN by activating AMPK/SIRT1/PGC-1*α* signaling in db/db mice.

## 4. Discussion

In this study, we provide new information regarding the role of GA as a treatment for DN. GA has been reported to perform a variety of biological and pharmacological activities, but the mechanism by which GA alleviates DN is not fully understood and requires further study [1]. Data presented in our previous studies demonstrate that GA exerts a protective effect against DN in high-glucose-stimulated HBZY-1 cells and db/db mice [17]. In this study, we further explored the molecular mechanisms underlying the protective effects that GA exerts against DN-induced oxidative stress damage.

Pharmacologically targeting transcriptional networks to regulate or modulate gene expression programs that favor energy expenditure is an attractive option for studies aimed at combating metabolic diseases, particularly those that lead to diabetic complications [18]. Because AMPK, SIRT1, and PGC-1*α* are involved in catabolism, mitochondrial activation, angiogenesis, and enhanced cell survival, they are important targets of interest [19–22].

However, the effects of GA on the kidneys of animal models of type 2 diabetes are not well understood. We therefore chose db/db mice as a model of type 2 DM. In this study, db/m mice were used as the control group. The experimental groups were treated with 15 mg/kg of GA, and the results showed that GA had no obvious effect on the blood glucose levels, body weight, and food intake in db/db mice. In contrast, Wang et al. [23] found that GA decreased blood glucose levels. This difference is potentially due to differences in the animal models and treatment methods used in these studies. In the Wang et al. study, male BALB/cA mice were used to build a model of diabetic nephropathy, and GA was administered to diabetic mice by mixing it with a powder diet. This disparity requires further investigation. However, GA clearly reduced Alb/crea levels in the mice in this study, and we therefore speculated that GA may alleviate renal injury caused by DN in the kidneys of db/db mice. HE and PAS staining and electron microscopy showed that after treatment with GA, the kidney damage observed in the db/db mice was significantly reduced, and the glomerular structure tended to be normal, consistent with the results reported in previous studies. In this study, we show that in db/db mice, DN is associated with an increase in the accumulation of renal lipids, apoptotic renal cell injury, and oxidative stress, all of which are related to the inactivation of AMPK/SIRT1/PGC-1*α* signaling. DN was ameliorated by treatment with GA, which activates AMPK/SIRT1/PGC-1*α* signaling.

In recent years, studies of pathological cases have shown that renal cells can express renal tissue fibrosis and that the transdifferentiation of muscle fibroblasts in the renal tissue may indirectly react to the degree of renal tissue fibrosis [24]. *α*-SMA is expressed in muscle fibroblasts, and mature renal cells are located only in the middle of the vessel wall. Presumably, GA inhibits *α*-SMA expression and thereby plays a role in slowing renal fibrosis. In addition, early diabetic glomerular and tubular hypertrophy play important roles in this process [25]. In our experiments, TGF-*β*1 protein levels were significantly higher in db/db mice than in mice in the other groups. This finding suggests that the cell hypertrophy observed in the db group was related to the high expression level of TGF-*β*1, which can block ECM degradation [26, 27]. Matrix metalloproteinases (MMPs) are enzymes that play key roles in substrate degradation. TGF-*β*1 inhibits the synthesis of MMP and the activation of fibrinolytic enzymes [28]. Additionally, it stimulates cell receptors and the ECM to trigger cells and induce interactions between the cells and the matrix, further exacerbating the accumulation of the ECM [29, 30]. In the present study, we verified these results in mice. In the experimental db/db group, *α*-SMA and TGF-*β*1 was present at higher levels than those observed in the normal group, and their expression was decreased after the addition of GA. Therefore, a method designed to prevent the expression of *α*-SMA and TGF-*β*1 might produce a protective effect against DN.

ROS can be generated during glucose metabolism in the mitochondria (via the electron transport chain (ETC) activity) and the plasma membrane (via NADPH oxidase (NADPHox)). But ROS are mainly produced by the mitochondria, during both physiological and pathological conditions [31]. The main antioxidant enzymes responsible for ROS removal are superoxide dismutase (SOD), glutathione reductase, glutathione peroxidase, and catalase. Evidence suggests that ROS are present in both db/db mice [32]. The importance of elevated ROS levels to the pathogenesis of microvascular complications in diabetes is well documented [33, 34]. Our results show that high glucose levels promote the generation of ROS and increase MDA and decrease SOD levels [17]. We further confirmed that high glucose levels induce ROS production, promote lipid peroxidation, and decrease antioxidant capacity. However, in the db + g group, ROS levels were decreased, and we observed that GA played an antioxidant role in these mice. A previous study showed that oxidative stress leads to lipid peroxidation as a result of the formation of harmful products by MDA [35]. In addition, the activities of antioxidant defense enzymes, which cause cellular damage, were decreased [36]. Finally, GA has been reported to play an antioxidant role in ameliorating carbon tetrachloride-induced liver injury [37].

It has been reported that AMPK/SIRT1/PGC-1*α* signaling is an important energy-sensing signaling pathway. It is important to note that AMPK and SIRT1 regulate each other and share many common target molecules, including PGC-1*α*. Moreover, AMPK and SIRT1 are clinically relevant in metabolic syndrome and type 2 diabetes because their effects on target molecules lead to mitochondrial dysfunction and oxidative damage [38]. SIRT1 also responds to changes in the availability of nutrients via a forkhead-dependent pathway [39] and PGC-1*α* [40]. A high level of expression of PGC-1*α* can increase ATP levels and reduce the production of ROS. Previous studies have shown that overexpressed PGC-1*α* effectively suppressed ROS production and apoptosis caused by oxidative stress [41]. Thus, evidence has shown that activating AMPK and SIRT1 induces the concurrent deacetylation and phosphorylation of their target molecules and decreases susceptibility to diabetes-associated disorders. The present study suggests that the GA-mediated activation of AMPK, SIRT1, and PGC-1*α* may prevent both the accumulation of renal lipids and cell injury. This in vivo data demonstrates that the diabetes-induced suppression of AMPK/SIRT1/PGC-1*α* signaling is activated by GA. These results suggest that AMPK and SIRT1 activate each other and jointly regulate their downstream effector PGC-1*α* in mesangial cells [42, 43]. High glucose levels can prevent mesangial cells from transitioning from G1 to S phase, reduce the proportion of cells in G1 phase, increase the proportion of cells in S phase, and promote DNA synthesis and cell proliferation [44]. Consistent with our results, several recent studies have demonstrated that adiponectin [45] or AICAR [46] protect renal podocytes and decrease albuminuria in diabetic animals by decreasing oxidative stress and apoptosis via a mechanism involving the activation of AMPK. However, GA prevents DN by directly scavenging ROS and blocks glucose-lipid metabolism via AMPK/SIRT1-independent mechanisms [47]. The discrepancy between our results and those reported in previous studies regarding AMPK/SIRT1 signaling might reflect differences in the dose of GA that was administered and the part of the renal tissue that was examined.

Our data support the conclusion that the effect of GA on DN is associated, in part, with the inhibition of ROS and the activation of AMPK/SIRT1/PGC-1*α* signaling in the kidneys of diabetic db/db mice. GA therefore shows potential as a therapeutic target for preventing and treating DN.
